# Efficacy of conbercept combined with panretinal photocoagulation in the treatment of proliferative diabetic retinopathy

**DOI:** 10.1038/s41598-020-65833-0

**Published:** 2020-05-29

**Authors:** Feng He, Jingyun Yang, Xiao Zhang, Weihong Yu

**Affiliations:** 1 Department of Ophthalmology, Peking Union Medical College Hospital, Chinese Academy of Medical Sciences, Beijing, China; 2 Key Laboratory of Ocular Fundus Diseases, Chinese Academy of Medical Sciences, Peking Union Medical College, Beijing, China; 3 Division of Statistics, School of Economics, Shanghai University, Shanghai, China; 4 Research Center of Financial Information, Shanghai University, Shanghai, China; 5 Rush Alzheimer’s Disease Center, Rush University Medical Center, Chicago, IL USA; 6 Department of Neurological Sciences, Rush University Medical Center, Chicago, IL USA

**Keywords:** Outcomes research, Retinal diseases

## Abstract

This prospective clinical study was to compare the effect of panretinal photocoagulation (PRP) associated with intravitreal conbercept injections versus PRP alone in the treatment of proliferative diabetic retinopathy (PDR). For each of 15 patients included, one eye was randomly assigned to receive treatment with PRP, and the other eye received conbercept combined PRP. Ophthalmic examinations, optical coherence tomography (OCT) and optical coherence tomography angiography (OCTA) were performed at baseline and at each monthly visit until 6 months. Fluorescein angiography (FA) was acquired at baseline, 3 months and 6 months. Between group and within group analysis was done by using generalized estimating equations (GEE). The combination group had a significant decrease of neovascularization (NV) leakage area than the PRP group at month 3 and month 6 after treatment, and a better best-corrected visual acuity (BCVA) during the first three months. Within-group analysis indicated a significant decrease in NV leakage at month 3 and month 6 in both groups, and a significant increase in BCVA at 1 month in the combination group. In summary, the combination of intravitreal injection of conbercept and PRP can significantly reduce the NV of PDR patients and achieve better BCVA during the drug’s lifespan compared with PRP alone.

## Introduction

Proliferative diabetic retinopathy (PDR) is the leading cause of severe vision loss in patients with diabetes worldwide^1^, and is characterized by retinal neovascularization (NV) at the disc (NVD) or elsewhere in the retina (NVE). Sight-threatening diabetic retinopathy affected approximately 12.6% of Chinese diabetic patients^2^. According to the American Academy of Ophthalmology’s latest Diabetic Retinopathy Clinical Guidelines in 2019^3^, panretinal photocoagulation (PRP) is recommended to be performed once NV appears. Meanwhile, intravitreal injection of anti-VEGF agents could also be used. Previous studies found that PRP combined with anti-VEGF agents such as ranibizumab was more effective for NV regression than PRP alone;^4–6^ but there was no unified scheme in different studies.

Conbercept is a recombinant fusion protein which consists of the 2nd Ig domain of VEGFR1 and the 3rd and 4th Ig domains of VEGFR2 combined with the constant region, i.e, Fragment of crystallizable (Fc) of human IgG1^7,8^. It has been approved by the Chinese Food and Drug Administration for the treatment of wet age-related macular degeneration, diabetic macular edema and choroid neovascularization secondary to pathologic myopia. However, there have been few reports of its off-label use for retinal NV in patients with PDR^9^.

In addition, examining the short-tern changes of retinal neovascularization to PRP or Conbercept may contribute to the determination of the efficacy of different treatment options and the optimal retreatment time. Therefore, we conducted this pilot study to investigate the efficacy and longitudinal changes in the NVE of PDR patients treated with PRP alone or intravitreal conbercept injection plus PRP.

## Methods

### Study participants

The study included a total of 15 consecutive patients who were treated at the Department of Ophthalmology of Peking Union Medical College Hospital from October 2017 to October 2018. We included patients diagnosed with treatment-naive high-risk PDR in both eyes as confirmed by fluorescein fundus angiography (FFA, Topcon Inc., Tokyo, Japan). Patients were excluded from the study if they had: 1) fibrovascular proliferation with retinal traction; 2) obvious optical media blurring affecting the evaluation of retina condition; 3) other causes of NV such as retinal vein occlusion; 4) atrophy, scarring, fibrosis, and hard exudates involving the central macula; or 5) a history of vitrectomy, optic neuropathy and uncontrolled glaucoma.

### Study design

For each patient, one eye was randomly assigned to receive treatment with PRP (the PRP group), and the other eye received conbercept combined PRP (the combination group). In the PRP group, PRP was performed in three sessions at a one-week interval according to the EDTRS guidelines^10^. Eyes in the combination group received one intravitreal injection of 0.5 mg/0.05 mL conbercept (Chengdu Kanghong Biotech Co., Ltd., Chengdu, Sichuan, China) twice, i.e., one week before PRP and one week after PRP.

All patients underwent comprehensive ophthalmic examinations, including ETDRS letters-measured best-corrected visual acuity (BCVA), intraocular pressure, slit-lamp biomicroscopy, indirect ophthalmoscopy, spectral domain optical coherence tomography (SD-OCT) and optical coherence tomography angiography (OCTA) examination. Data were recorded at baseline and during monthly visits until 6 months. FFA was obtained at baseline, month 3 and month 6.

Digital fundus fluorescein angiography of ETDRS 7-standard field was obtained using a 50° fundus camera system (TRC-50X/IMAGEnet; Topcon, Tokyo, Japan)^11^. The total area of fluorescein leakage from active NVs at 1 minute was measured in mm^2^. If there was more than one site of active NVs, all sites were considered for analysis. When no leakage was observed on FFA images, complete NV regression was considered. Macular scan protocol (512 × 128 mode) was performed using a SD-OCT device (Topcon Inc., Tokyo, Japan). Central retinal thickness (CRT) was calculated as the average thickness of a central macular area with a diameter of 1 mm, centered on the fovea of the patient. When CRT was above 300 μm, diabetic macular edema (DME) was considered. OCTA images were acquired with the RTVue-XR Avanti system (Optovue Inc., Fremont, California, USA) using “HD Angio Retina 6 × 6 mm” mode, foveal avascular zone (FAZ), and superficial retina capillary flow density (FD) was automatically measured.

### Statistical analysis

We examined the following outcomes regarding whether there was a difference in changes from baseline between the two treatment groups: NV leakage area, total regression rate of NV, BCVA, CRT, FAZ, and FD. We used generalized estimating equations (GEE) to take into account the correlation of changes over time by including a term for time and the main effect of treatment, and a term for the interaction between them. Using a similar approach, we also performed within-group comparisons of these outcomes regarding their changes from baseline to examine the efficacy of each individual treatment regime. All statistical analyses were performed using SAS version 9.4 (SAS Institute, Inc., Cary, NC, USA). P < 0.05 was considered statistically significant. This study was approved by the institutional review board of Peking Union Medical College Hospital and adhered to the tenets of the Declaration of Helsinki. Informed consent was obtained from all subjects.

## Results

A total of fifteen patients were included in this study. Patients’ characteristics are summarized in Table 1. Their mean age was 47.7 ± 11.6 years with a median DM history of 15.2 ± 7.1 years and mean glycated haemoglobin (HbA1C) 7.9% ± 1.2%. There were 5 eyes with mild cataract, and 2 eyes underwent cataract extraction more than one year ago in each group. Basic ocular characteristics of the two groups are summarized in Table 2; there was no significant difference in these variables between two groups. No suspected unexpected serious adverse reactions were observed during the study. No significant increase in intraocular pressure was observed during the study period.

**Table 1:** Basic characteristics of the study participants.

Age (years)	47.7 ± 11.6
Gender (female)	8 (53.3%)
Duration of DM (years)	15.2 ± 7.1
Mean HbA1c (SD), %	7.9 ± 1.2
Insulin users	12 (80.0%)
DME (eyes)	6 (20.0%)
History of eye diseases (eyes)	10 (33.3%)
History of eye surgery (eyes)	4 (13.3%)
Hypertension	3 (20.0%)

DME, diabetic macular edema; DM, diabetes mellitus; SD, standard deviation.

**Table 2:** Basic ocular characteristics of the two groups.

	PRP group	Combination group	*P*
DME (eyes)	3	3	—
mean BCVA (ETDRS)	46.93 ± 18.73	40.87 ± 18.76	0.383
mean CRT (μm)	268.5 ± 122.3	277.5 ± 120.7	0.841
mean leakage area (mm^2^)	9.464 ± 6.762	14.879 ± 8.443	0.063
mean FAZ (mm^2^)	0.335 ± 0.095	0.333 ± 0.116	0.965
mean FD (%)	46.35 ± 4.21	45.24 ± 5.43	0.548

PRP, panretinal photocoagulation; DME, diabetic macular edema; BCVA, best-corrected visual acuity; ETDRS, the Early Treatment Diabetic Retinopathy Study; CRT, central retinal thickness; FAZ, foveal avascular zone; FD, flow density.

**Figure 1:**
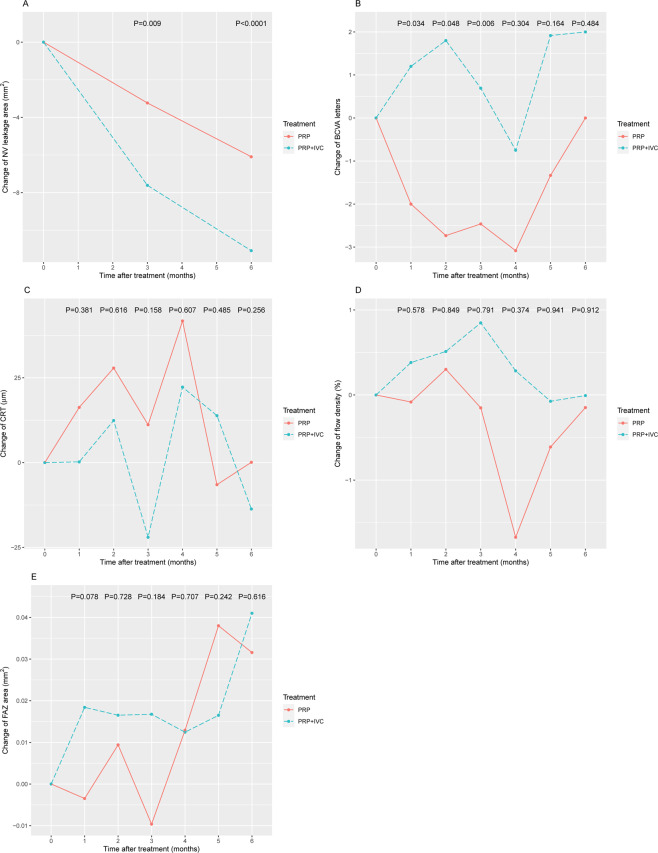
Changes between the combination group (PRP + IVC) and the PRP group. **(A)** NV leakage area; **(B)** BCVA; **(C)** CRT; **(D)** flow density; and **(E)** FAZ. Comparisions were made using generalized estimating equations (GEE) to take into account the correlation of changes over time. PRP, panretinal photocoagulation; IVC, intravitreous conbercept; NV, neovascularization; BCVA, best-corrected visual acuity; CRT, central thickness; FAZ, foveal avascular zone.

GEE analysis indicated that patients in the combination group had a significant decrease, compared with the baseline, in NV leakage area than the PRP group at month 3 (−7.61 vs. −3.24 mm^2^; P = 0.0009) and month 6 after treatment (−11.10 vs. −6.10 mm^2^; P < 0.0001; Fig. 1A). Based on FA results, neither group demonstrated complete NV regression at month 3, while the complete NV regression rate at both groups was 13.3% (2/15 eyes) at month 6. During the first 3 months after treatment, patients in the combination group had a significant increase in BCVA than those in the PRP group (month 1: 1.20 vs. −2, P = 0.034; month 2: 1.80 vs. −2.73, P = 0.048; and month 3: 0.69 vs. −2.46, P = 0.006; Fig. 1B). We did not observe a significant difference in the change of CRT (Fig. 1C), FD (Fig. 1D) and FAZ (Fig. 1E) between the two groups at any time point.

**Figure 2:**
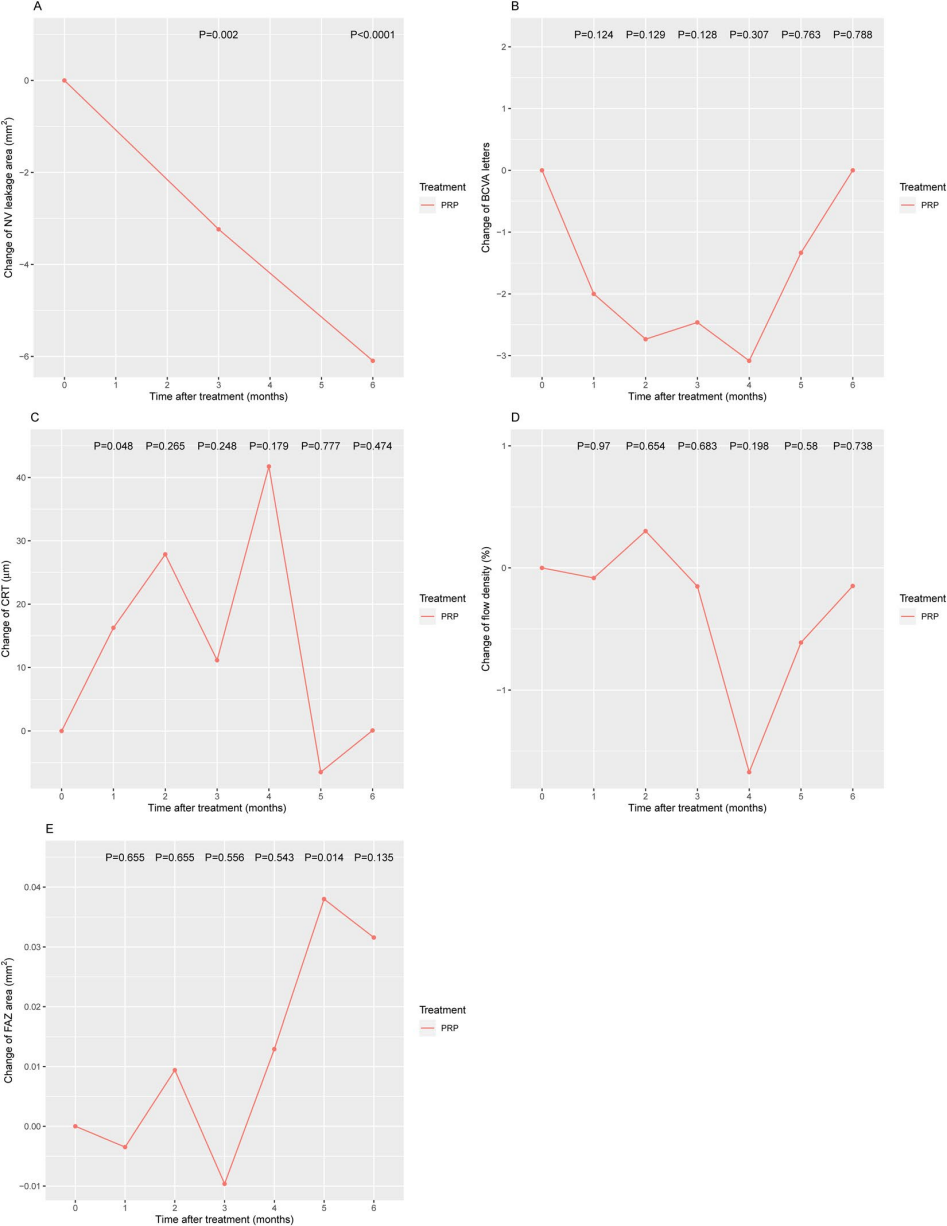
Changes in the PRP group. (**A**) NV leakage area; (**B**) BCVA; (**C**) CRT; (**D**) flow density; and (**E**) FAZ. Comparisions were made using generalized estimating equations (GEE) to take into account the correlation of changes over time. PRP, panretinal photocoagulation; IVC, intravitreous conbercept; NV, neovascularization; BCVA, best-corrected visual acuity; CRT, central thickness; FAZ, foveal avascular zone.

Within-group analysis of PRP treatment indicated a significant decrease, compared with the baseline, in NV leakage at month 3 and month 6 after treatment (month 3: −3.24, P = 0.002; month 6: −6.10, P < 0.0001; Fig. 2A). We observed a significant increase in CRT at 1 month after treatment (change=16.30, P = 0.048; Fig. 2C) and a significant increase in FAZ at month 5 after treatment (change=0.038, P = 0.014; Fig. 2E). We did not observe a significant difference in the change of BCVA (Fig. 2B) and FD (Fig. 2D).

**Figure 3:**
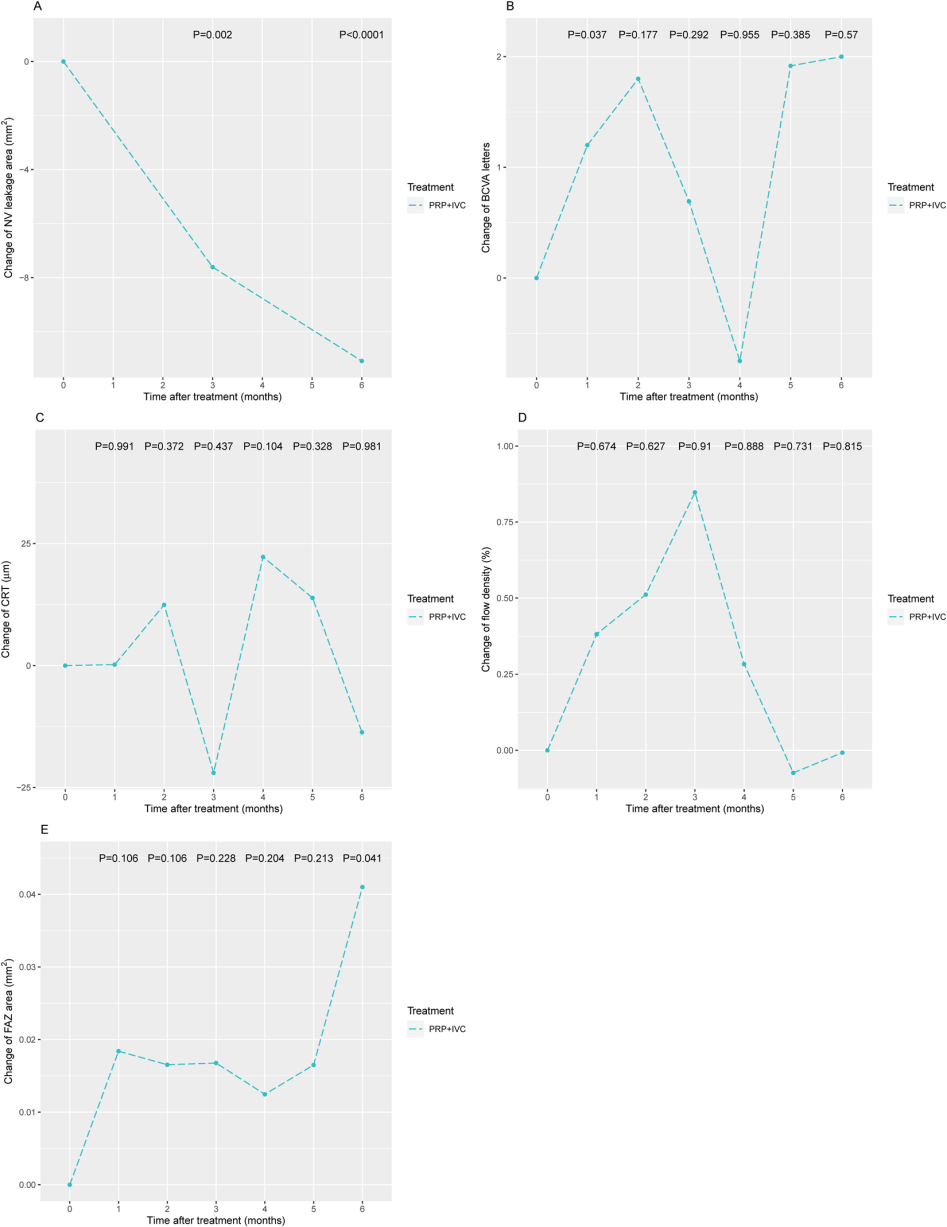
Changes in the combination group (PRP + IVC). (**A**) NV leakage area; (**B**) BCVA; (**C**) CRT; (**D**) flow density; and (**E**) FAZ. Comparisions were made using generalized estimating equations (GEE) to take into account the correlation of changes over time. PRP, panretinal photocoagulation; IVC, intravitreous conbercept; NV, neovascularization; BCVA, best-corrected visual acuity; CRT, central thickness; FAZ, foveal avascular zone.

Within-group analysis of the combination treatment also indicated a significant change, compared with the baseline, in NV leakage at month 3 and month 6 after treatment (month 3: −7.61, P = 0.002; month 6: −11.1, P < 0.001; Fig. 3A). We observed a significant increase in BCVA at 1 month after treatment (change=1.2, P = 0.037; Fig. 3B), and a significant increase in FAZ at month 6 after treatment (change=0.041, P = 0.041; Fig. 3E). We did not observe a significant difference in the change of CRT (Fig. 3C) and FD (Fig. 3D).

## Discussion

In this study, we compared the clinical effect of twice intravitreal injections of conbercept with traditional PRP regimen. Our results demonstrated that both treatment regimens significantly reduced the area of NV leakage. However, patients in the combined group had significantly reduced NV leakage area based on FA and improved BCVA at one month after the treatment than the PRP group. In contrast, patients in the PRP group had increased CRT at one month. Therefore, the combined therapy could be a potentially favorable treatment therapy for high-risk PDR.

In the past four decades, PRP has been the standard treatment for PDR. It can induce the regression of NV and reduce the risk of severe vison loss^12^. The primary treatment target is NV regression to prevent further vitreous hemorrhage. A recent study showed that with PRP alone, 78.9% cases showed partial regression and the mean NV area was reduced by 30.6% at 3 months. At one year, compared with baseline, 25% of eyes had total regression, 62.5% had partial regression and 12.5% had no regression, with a mean reduction of NV area by 75.5%^13^. In another study, the mean NV area decreased by 32.9% at month 2 after PRP treatment^14^.

Anti-VEGF drugs have been shown to effectively attenuate NV^15,16^. However, protocol S requires 6 consecutive injections, resulting in heavy financial and clinical follow-up burden to a patient. Our findings indicated that PRP combined with anti-VEGF could be a better choice for developing countries such as China in that the combination therapy could more effectively eliminate NV without the need of 6 consecutive injections.

In this study, we found no significant difference in CRT at all time points between the two groups. However, in the PRP group, CRT increased significantly at 1 month after treatment completion, and then returned to baseline levels, indicating that PRP treatment may cause short-term macular edema, although it did not necessarily affect BCVA, similar to the findings by Soman *et al*.^17^. This maybe because the impact was relatively mild and patients could recover faster.

Compared with the PRP group, patients in the combination group experienced significant BCVA increases within the first three months after the treatment, indicating that anti-VEGF treatment improved visual function. However, the significance in difference between the two groups disappeared toward the end of the study. We may not have sufficient statistical power to detect the difference in the later follow-up period. Moreover, most patients in our study may not have significant macular edema at baseline, and therefore, the increase in BCVA after treatment with regressive neovascularization was not significant.

We did not observe a significant difference in the change of FD and FAZ between the two groups. Although neither treatment regimen showed benefit with respect to FD and FAZ at each time point, in intra-group analysis we found a significant increase in FAZ at month 5 after treatment in the PRP group and a significant increase in FAZ at month 6 after treatment in the combination group, suggesting that both treatment regimens did not stop the potential progress of macular ischemia.

The complete regression rate of NV was the same at 6 months in both groups (13.3%, 2/15 eyes), and the combination group showed no superiority. This indicated that PRP may need to be combined with more anti-VEGF injections to completely resolve NV. Further research is needed on the best combination treatment strategy.

There are some limitations in this study. The sample size is relatively small and we may not have sufficient statistical power for some of the analyses. The 6-month follow-up period is relatively short, preventing us to compare the longer-term effect between the two groups. Further randomized studies with larger sample sizes and longer follow up will enhance the results obtained in this study.

In conclusion, we observed that the combination of intravitreal injection of conbercept and PRP could significantly reduce the NV of PDR patients and achieve better BCVA during the drug’s lifespan compared with PRP alone. However, it is possible that PRP combined with more anti-VEGF injections can achieve better results.
